# Cetirizine use in childhood: an update of a friendly 30-year drug

**DOI:** 10.1186/s12948-020-00118-5

**Published:** 2020-02-26

**Authors:** Giuseppe Fabio Parisi, Salvatore Leonardi, Giorgio Ciprandi, Angelo Corsico, Amelia Licari, Michele Miraglia del Giudice, Diego Peroni, Carmelo Salpietro, Gian Luigi Marseglia

**Affiliations:** 1 Department of Clinical and Experimental Medicine, University of Catania, Via Santa Sofia, 78, 95123 Catania, Italy; 2 Allergy Clinic, Casa di Cura Villa Montallegro, Genoa, Italy; 3 Pulmonology Clinic, Foundation IRCCS Policlinico San Matteo, University of Pavia, Pavia, Italy; 4 Department of Pediatrics, Foundation IRCCS Policlinico San Matteo, University of Pavia, Pavia, Italy; 5 Department of Woman, Child and of General and Specialized Surgery, University of Campania Luigi Vanvitelli, Naples, Italy; 6 U.O. Pediatria, Azienda Ospedaliero-Universitaria Pisana, Scuola di Specializzazione in Pediatria, University of Pisa, Pisa, Italy; 7 Unit of Pediatric Genetics and Immunology, Department of Pediatrics, University of Messina, Messina, Italy

**Keywords:** Cetirizine, Antihistamines, Histamine, H1 receptors, Children, Adolescents, Pregnancy, Lactation

## Abstract

Cetirizine is a second-generation antihistamine, derived from the metabolism of hydroxyzine, highly specific for the H1 receptors, and with marked antiallergic properties. Although its history began more than 30 years ago, it remains one of the most used drugs in children with a leading role in the medical care of children with allergic diseases. Cetirizine use is licensed for paediatric patients for the treatment of allergic rhinitis, and chronic spontaneous urticaria, in Europe in children older than 2 years old and in the USA in children older than 6 months old. This review provides a practical update on the use of cetirizine in children and adolescents.

## Background

Cetirizine is a second-generation antihistamine derived from the metabolism of hydroxyzine, with high specificity for the H1 receptors and antiallergic properties [1]. Although its history began more than 30 years ago, it remains one of the most used drugs in children, accounting for about 9% of all paediatric prescriptions [2]. Cetirizine is an orally administered drug that reaches a peak concentration about 2 h after its administration in children; it is then only slightly metabolized in the liver and then eliminated by renal excretion [3]. In children, the cetirizine half-life is reduced because of their increased hepatic metabolism; this may justify the occasional need to double the daily dose. Adolescents could take cetirizine once a day, as well as adults [1]. Cetirizine exceeds the blood–brain barrier in minimal quantities and, for this reason, does not cause the classic sedative effects associated with other antihistamines [4]. In allergic subjects, the drug acts to antagonize the secretion of histamine, inhibit the recruitment of eosinophils, release leukotriene B4, and decrease the expression of the vascular cell adhesion molecule (VCAM-1), thus also exerting a powerful anti-inflammatory effect [3].

Nowadays, cetirizine use is licensed for children and adolescents for the treatment of allergic rhinitis and chronic spontaneous urticaria (CSU); children should be older than 2 years old, although the US Food and Drug Administration (FDA) licensed cetirizine also for children aged over 6 months [5, 6]. Cetirizine is administered at the dose of 0.25 mg/kg/day (1 drop = 0.5 mg) [5].

This review summarizes the evidence regarding the 30-year-history of cetirizine. The use of the drug during pregnancy and lactation will also be evaluated.

## Safety profile

Antihistamines, especially first-generation ones, have central effects that cause drowsiness, tiredness, increased appetite, or worsening of cognitive functions (Table 1). They may also have antimuscarinic, antiadrenergic, and antiserotoninergic activity that may cause vision disorders, dry mouth, tachycardia, and confusion [7]. The use of first-generation antihistamines that have sedative effects is not anymore recommended in children. Some antihistamines, particularly ebastine and mizolastine, can cause a significant prolongation of the QT interval on an electrocardiogram, which can cause severe arrhythmias, even life-threatening, such as torsade de point [8].

**Table 1:** Most common first and second-generation antihistamines

First generation	Second generation
Diphenhydramine	Cetirizine
Clemastine	Levocetirizine
Clorphenamine	Ketotifen
Dimetindene	Loratadine
Hydroxyzine	Desloratadine
Promethazine	Rupatadine
Alimemazine	Ebastine
Cyproheptadine	Bilastine
	Fexofenadine

An original report published in 2017 showed that the use of antihistamines is associated with severe and unexpected adverse drug reactions (ADRs), and 400 patients had a fatal outcome. More than half of deaths (205 cases) involved children under 2 years of age, and 74 of them involved suspected drugs not licensed in that age‐group. One of the most significant points that was highlighted was the finding that serious ADRs had been correlated with off-label use concerning the indication, age group, and overdose [9].

Cetirizine appears to be well tolerated in children. The most common side effects (e.g., drowsiness, headache, pharyngitis, abdominal pain, diarrhea, coughing) do not seem to be more frequent in clinical trials that have evaluated the efficacy and safety of cetirizine compared to placebo or other antihistamines [10–12]. The tolerability of the drug is comparable also to the newer anti H1, for example bilastine or levocetirizine [13]. Regarding somnolence, this appears to be dose-related, with an incidence of approximately 2–4% of patients compared to 1% of placebo patients. Interestingly, children tolerate better this adverse effect than adults [1]. Furthermore, cetirizine does not appear to be associated with significant cardiac alterations such as changes in the QT interval in pediatric populations [14], and even doses 4 times higher than the recommended dose have not been associated with significant toxicity affecting the heart or central nervous system [15]. Finally, recently, a new topical ophthalmic solution (0.24%) was approved by the FDA for the treatment of allergic conjunctivitis with a demonstrated safety profile in children older than 2 years old [6].

## Conventional clinical indications

### Allergic rhinitis and conjunctivitis

Histamine is the most potent mediator that is released during the early phase of an allergic reaction and causes itching, sneezing, rhinorrhoea, and nasal congestion. The Allergic Rhinitis and its Impact on Asthma (ARIA) guidelines recommend using second-generation oral antihistamines for both seasonal (SAR) and perennial (PAR) rhinitis; these molecules represent the most suitable preparations to treat allergic rhinitis because of their already mentioned ‘antiallergic-anti-inflammatory’ activity and excellent safety profile. Such drugs can be used as needed if the symptoms are occasional. In SAR, treatment with anti-H1 antihistamines could be started before exposure to the allergens to downregulate constitutive H1 receptor activity and shift the equilibrium from the active form of the H1 receptor to the inactive form, thus preventing the massive release of histamine by mast cells; this treatment has to be continued for the entire duration of pollination. In PAR, the treatment should instead be modulated based on clinical symptoms, and it has the dual purpose of controlling the persistent mucosal inflammation that reduces the inflammatory mucosal infiltrate and adhesion molecule expression [16, 17].

Among antihistamines, cetirizine has a relatively higher affinity and selectivity for H1 receptors, which confers a more potent, faster onset, and longer duration of action with even anti-inflammatory properties independent of its anti-H1 effects [18, 19]. Ciprandi et al. [20] demonstrated that cetirizine exerts a significant anti-inflammatory activity in children with PAR decreasing the nasal levels of interleukin (IL)-4 and IL-8. Similar results were obtained by Uguz and colleagues [21], who demonstrated that cetirizine induces a shift in the Th1/Th2 cytokine balance towards a Th1 response, increasing the production of interferon (IFN)-gamma and IL-10. From a clinical point of view, cetirizine was shown to be more effective when compared with placebo in the treatment of SAR with a significant reduction in the number of days characterized by the absence of symptoms assessed by questionnaires [14, 22, 23]. Furthermore, the efficacy of cetirizine appears to be immediate (within 24 h) and lasting over time when compared with first-generation antihistamines and is not burdened by significant side effects [24]. Also, when compared with other second-generation antihistamines (see Table 1), cetirizine seems to be more effective: Lee et al. showed that the 12-week treatment program with cetirizine was more effective than levocetirizine [25], and recently, in a randomized, placebo-controlled study, a better effect of cetirizine was found in the treatment of allergic rhinitis when compared with loratadine [26]. Concerning PAR, the effect of cetirizine has also been compared to montelukast. Although both products appear to be effective in reducing symptoms compared to placebo, cetirizine offers better results in improving nasal itching [27]. Furthermore, clinical studies demonstrate the importance of long-term cetirizine therapy: in children with dust mite allergy, treatment for at least 6 months results in a significant reduction in the prescription of other drugs (e.g., inhaled corticosteroids, beta2-agonists, antibiotics) compared to placebo [28]. In the same category of children, it has been shown that treatment for at least 3 years with cetirizine is associated with a reduction of new allergic sensitizations [29].

Regarding dosage, it was confirmed that 10 mg per day in children over 6 years (divided into two administrations if less than 12 years) is better when compared with 5 mg in terms of symptom reduction, both concerning rhinitis and conjunctivitis. The administration of 5 mg is only useful for the reduction of sneezing [30]. Table 2 summarizes the evidences on the efficacy of cetirizine in the treatment of allergic rhinitis and conjunctivitis.

**Table 2:** Summary of evidence on the efficacy of cetirizine in the treatment of allergic rhinitis

References	Disease	Subjects	Main results
Ciprandi et al. [20]	Perennial allergic rhinitis	20 children	Decreasing of nasal IL-4 and IL-8 levels
Uguz et al. [21]	Perennial allergic rhinitis	13 children	Increasing the production of IFN-gamma and IL-10
Allegra et al. [22]	Seasonal allergic rhinitis	107 children aged 2–6 years	Improvement in sneezing, rhinorrhea, nasal obstruction, nasal and ocular pruritus than placebo
Masi et al. [23]	Seasonal allergic rhinitis	124 children aged 6–12 years	Improvement in sneezing, rhinorrhea, nasal obstruction, nasal and ocular pruritus than placebo
Pearlman et al. [14]	Seasonal allergic rhinitis	209 children aged 6–11 years	Improvement in sneezing, nasal discharge, itchy eyes, itchy nose or mouth, conjunctivitis, nasal congestion than placebo
Nayak et al. [25]	Seasonal allergic rhinitis	683 children aged 6–11 years	Improvement in TSSC than loratadine and placebo
Lee et al. [26]	Perennial allergic rhinitis	74 children aged 6–12 years	Improvement in TSS than levocetirizine and placebo
Chen et al. [27]	Perennial allergic rhinitis	60 children aged 2–6 years	Improvement in eosinophil percentage in nasal smears, PRQLQ and TSS. Cetirizine better than montelukast for itching
Ciprandi et al. [28]	Rhinitis and/or mild asthma	10 children	Improvement in symptoms and reduction of drug consumption than placebo
Ciprandi et al. [29]	Mite allergy	20 children	Lower incidence of new sensitisations
Pearlman et al. [30]	Seasonal allergic rhinitis	209 children aged 6–11 years	Improvement in TSS than placebo

*TSSC* total symptom score complex, *TSS* total symptom score, *PRQLQ* paediatric rhinoconjunctivitis quality of life questionnaire

### Chronic spontaneous urticaria (CSU)

Anti-H1 antihistamines are effective at reducing itchiness number, size, and duration of wheals and erythema in patients with acute or chronic urticaria. The current guidelines recommend the use of second-generation molecules for their tolerability and safety profile. In CSU, progressively increasing the dose of second-generation H1-antihistamines up to fourfold higher than the recommended dose, or using a combination of two different second-generation antihistamines, is suggested when there is treatment failure using standard doses [31, 32].

A double-blinded, multicentre study performed on 62 children aged 2–6 years showed that cetirizine was safe and effective in the treatment of urticaria symptoms (erythema, papules, edema, itching) when compared with oxatomide [12]. Furthermore, cetirizine seems to be effective in 12–24 month-old children with atopic dermatitis in the prevention of acute urticaria [33]. A recently published case report showed a successful treatment of severe CSU in a 14-year-old boy who was supposed to be unresponsive to omalizumab since its CSU was not IgE-mediated with conventional treatment (cetirizine, montelukast, systemic steroids, and dietary restriction) [34]. Regarding the dose to be administered, already Komeyoshi et al. [35] had shown that in patients who had responded poorly to therapy with the initial dosage, doubling the dose of cetirizine resulted in improved symptom control. These findings were confirmed later by Okubo et al. [36] in a prospective, randomized, non-blinded study they showed that doubling the cetirizine dose led to an improvement in the quality of life and severity of wheal and itching in 64.7% of treated patients. Recent data support the safety of the drug even when the dose is up to fourfold or prolonged administered [37, 38].

## Unconventional uses

### Atopic eczema

Although their use is controversial, antihistamines are commonly used in the treatment of itching associated with atopic dermatitis. The National Institute for Health and Care Excellence (NICE) guidelines for treating atopic eczema suggest a 1-month trial of a non-sedating antihistamine in children with severe itching; this treatment may be continued, if successful, while symptoms persist, but this treatment regimen should be reviewed every 3 months [39].

The historical double-blinded, randomized, placebo-controlled ETAC trial evaluated the efficacy of cetirizine in children with atopic dermatitis in the prevention of asthma. They showed that cetirizine delays or prevents the development of asthma in children with atopic dermatitis sensitized to grass pollen (RR = 0.5, p = 0.002) and, to a lower degree (RR = 0.6, p = 0.005), house dust mites when compared to placebo-treated controls [40]. In the same cohort of patients, the consumption of drugs for the same conditions was studied, demonstrating that other oral H1-antihistamines were significantly more often used in the placebo group than in the cetirizine group (24.9% vs. 18.6%, p = 0.03). Moreover, cetirizine reduced the amount and duration of moderate-to-strong topical corticosteroids needed to treat subjects with atopic eczema [41]. Recently, a Cochrane review evaluated the efficacy of oral H1 antihistamines as an add-on therapy to topical treatment for eczema, highlighting how, despite the scientific evidence being qualitatively limited, cetirizine was burdened by fewer side effects and less need for additional H1-antihistamines in case of eczema flare compared with other antihistamines used as an add-on therapy [42].

### Asthma

Antihistamines should not be considered a therapeutic option in case of asthma but may have a role in treating comorbidities such as allergic rhinitis and in that way exert an indirect positive impact on asthma control. In fact, during allergic rhinitis and asthma, the upper and lower airways are affected by a common inflammatory process that can be sustained and amplified by interconnected mechanisms. Allergic rhinitis and non-specific vasomotor rhinitis are some of the most critical risk factors for the onset of asthmatic disease, and they are therefore important aggravating factors [43]. Thus, therapy with anti-H1 antihistamines confers an additional benefit in the control of asthmatic symptoms in subjects with concomitant allergic rhinitis and bronchial asthma [44].

Studies that evaluated the efficacy of cetirizine in patients with mild or moderate asthma were performed in adult populations in which allergic rhinitis coexisted. These studies, many of which were randomized and placebo-controlled, showed that doses ranging from 10 to 30 mg of cetirizine determine an improvement in asthma symptoms (but not always in pulmonary function tests), especially when the treatment reached 5–6 weeks [45–49].

## Cetirizine during pregnancy and lactation

It has been observed that about 25% of pregnant women suffer from allergic diseases, mainly urticaria, and rhinoconjunctivitis, which need to be treated with antihistamines to reduce symptoms and improve quality of life. Already in 2008, Weber-Schoendorfer and Shaefer evaluated a cohort of 196 pregnant women and showed that exposure to cetirizine in the first trimester of pregnancy was not associated with an increased risk of abortions or fetal malformations [50]. These data on safety have also been confirmed recently in observational cohort studies and meta-analyses [51, 52].

Regarding breastfeeding, a study conducted with telephone interviews reported that breastfed babies whose mothers took antihistamines were irritable, but none of these symptoms were relevant, so no medical consultation was necessary [53]. The British Society for Allergy and Clinical Immunology recommends cetirizine as an antihistamine of choice in the case of breastfeeding at the minimum possible dose and for the shortest possible time. In fact, elevated doses or prolonged use can cause excessive drowsiness or, on the contrary, excessive irritability in breast-fed children, especially if they are administered concomitantly with sympathomimetics such as pseudoephedrine [54].

## Conclusions

Cetirizine is a second-generation antihistamine that retains a leading role in the effective treatment of children with allergic diseases. The evidence of 30-year-clinical-experience support this drug above all as the first choice for good safety data during pregnancy and breastfeeding.

## Data Availability

Not applicable.

## Abbreviations

ADRs: Adverse drug reactions
ARIA: Allergic Rhinitis and its Impact on Asthma
CSU: Chronic spontaneous urticarial
FDA: Food and Drug Administration
IFN: Interferon
IL: Interleukin
NICE: National Institute for Health and Care Excellence
PAR: Perennial allergic rhinitis
SAR: Seasonal allergic rhinitis
TSSC: Total symptom score complex
VCAM-1: Vascular cell adhesion molecule
